# Direct repeats found in the vicinity of intron splice sites

**DOI:** 10.1007/s00114-025-01966-4

**Published:** 2025-01-30

**Authors:** Scott O. Rogers, Arnold J. Bendich

**Affiliations:** 1 Department of Biological Sciences, Bowling Green State University, Bowling Green, OH 43403 USA; 2 Department of Biology, University of Washington, Seattle, WA 98195 USA

**Keywords:** Group I, Group II, Spliceosomal, Archaeal, Selfish DNA

## Abstract

Four main classes of introns (group I, group II, spliceosomal, and archaeal) have been reported for all major types of RNA from nuclei and organelles of a wide range of taxa. When and how introns inserted within the genic regions of genomes, however, is often unclear. Introns were examined from Archaea, Bacteria, and Eukarya. Up to 80 bp surrounding each of the 5' and 3' intron/exon borders were compared to search for direct repeats (DRs). For each of the 213 introns examined, DNA sequence analysis revealed DRs at or near the intron/exon borders, ranging from 4 to 30 bp in length, with a mean of 11.4 bp. More than 80% of the repeats were within 10 bp of the intron/exon borders. The numbers of DRs 6–30 bp in length were greater than expected by chance. When a DNA segment moves into a new genomic location, the insertion involves a double-strand DNA break that must be repaired to maintain genome stability and often results in a pair of DRs that now flank the insert. This insertion process applies to both mobile genetic elements (MGEs), such as transposons, and to introns as reported here. The DNA break at the insertion site may be caused by transposon-like events or recombination. Thus, introns and transposons appear to be members of a group of parasitic MGEs that secondarily may benefit their host cell and have expanded greatly in eukaryotes from their prokaryotic ancestors.

**Supplementary Information:**

The online version contains supplementary material available at 10.1007/s00114-025-01966-4.

## Introduction

Cellular genes contain DNA sequences that encode RNA and protein products. In order to benefit the cell, those sequences typically must be contiguous and uninterrupted by extraneous DNA sequences that would compromise function. Introns, however, do separate the functional parts of genes and are typically removed from the RNA transcripts allowing the RNA to contribute to cellular activities. Introns are present in all domains of life and are identified by comparing initial transcripts with the mature RNAs, by comparing the intron-containing gene with the same gene lacking the intron, or by comparing the DNA sequence of the gene with the mature RNA product (Gilbert 1978; Shivji et al. 1996; Shinohara et al. 1996; Perotto et al. 2000; Harris and Rogers 2008; Jo and Choi 2015; Randau and Söll 2008; Rogers 2019a). The fact that one strain of a species can carry an intron that is absent from another strain, or an intron is present in one member of a gene family but absent from another member (Collins and Lambowitz 1983; Jacquier and Dujon 1985; Salvo et al. 1998), indicates that introns were mobile in the past and may still be mobilized, a property shared with mobile genetic elements (MGEs), including transposons. Whereas the evolutionary relationships among non-LTR retrotransposons, group II introns, and spliceosomal introns have been described, primarily for mRNA genes (Zimmerly et al 1995; Singh 2002; Aizawa et al. 2003; Roy 2004; Lambowitz and Zimmerly 2011; Yenerall and Zhou 2015; Zimmerly and Semper 2015; Novikova and Belfort 2017; Monachello et al. 2021; Gozashti et al. 2022; Roy et al. 2023; Wilkinson et al. 2023), the relationships inclusive of all transposons and introns (e.g., DNA transposons, group I introns, and archaeal introns), including their insertion mechanisms into tRNA, rRNA, and mRNA genes (transfer RNA, ribosomal RNA, and messenger RNA, respectively), have remained unresolved.

**Fig. 1:**
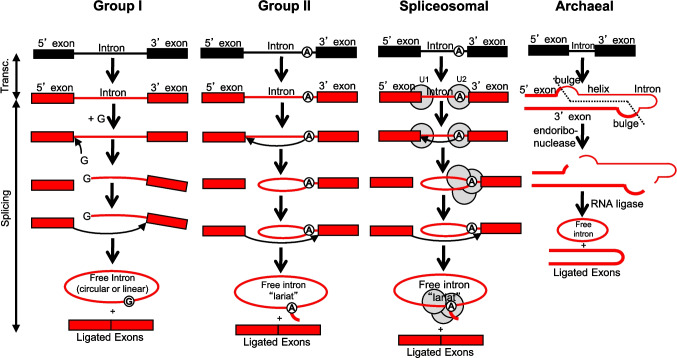
Splicing of intron types. Group I introns are self-splicing, in that they are ribozymes containing sequences that can cause cleavage and ligation reactions. Two transesterification reactions occur. The first is a nucleophilic attack on the 5' splice border by the 3' hydroxyl of the free guanosine (G) held in the intron pairing region P7, which separates the 5' exon from the intron, leaving the G covalently bonded to the 5' end of the intron. The second nucleophilic attack is initiated by the 3' hydroxyl at the 3' end of the 5' exon on the 3' intron/exon border. This event covalently joins the two exons and separates the 3' exon from the intron, resulting in a free linear intron that then typically circularizes. Group II introns also are self-splicing, and use a similar splicing pathway, except that the initiator of the first reaction is an adenine (A) near the 3' end of the intron. As with group I introns, there are two transesterification reactions. In the first, the nucleophilic attack by the 2' hydroxyl on the A within the intron separates the 5' exon from the intron, and the intron forms a lariat link with bonds to the 2', 3', and 5' carbons. The second reaction is a nucleophilic attack similar to that for group I introns, which results in joined exons and a free lariat. Group III introns (not shown) are a subtype of group II introns. Twintrons (not shown) are introns within introns, and have been described for groups I, II, and III (Hafez and Hausner 2015). The interior intron is removed prior to the removal of the exterior intron resulting in splicing of the surrounding exons. Spliceosomal introns undergo the same reactions as those for group II introns, but they are held in their reactive form by spliceosomes, which are small ribonuclear protein structures (Singh 2002). Some of the small RNAs in spliceosomes are similar to regions in the longer group II introns, and at least one of the proteins has similarities to ribosomal proteins. Introners are mobile spliceosomal introns and splice in a similar manner. Archaeal introns are first cut at two bulges within the splice sites (a bulge-helix-bulge structure, B-H-B) by an endoribonuclease, and an RNA ligase joins the ends of the exons as well as ligating the ends of the exon to form a circular RNA (Yoshihisa 2014). A similar set of reactions occurs when the large and small rRNA subunits are cleaved from a precursor RNA during maturation of the rRNAs. Some group I introns among Archaea also splice using a B-H-B mechanism, thus linking group I and archaeal introns evolutionarily

Introns have been classified according to their modes of splicing (Fig. 1). Groups I and II are found in Bacteria and Archaea, and Archaea have an additional archaeal type of intron present in some tRNA, rRNA, and mRNA genes (Yoshihisa 2014). Many of the archaeal introns are also found in tRNA genes in nuclei, mitochondria, and plastids. Whereas the number of introns is limited in prokaryotes, their number and diversity have expanded greatly in eukaryotes, including multiple introns within individual genes and the use of alternative splicing and trans-splicing, increasing the sizes of genomes and proteomes (Kumari et al. 2022). This expansion has led to the increase of protein diversity without requiring the addition of new genes. Therefore, introns have been retained due to their occasional beneficial effects.

Eukaryotic genomes contain not only group I, group II, and archaeal introns, but also spliceosomal introns, group III introns, and twintrons. Group II introns are the ancestors of spliceosomal introns (requiring spliceosomes) and group III introns (Chalamcharla et al. 2010; Yoshihisa 2014; Novikova and Belfort 2017). Twintrons are introns within other introns and have been documented for groups I, II, and III (Hafez and Hausner 2015). Introns have not only been beneficial in expanding gene and protein diversity but have added flexibility in gene expression (Sironi et al. 2002; Kakka et al. 2018; Marasco and Kornblihtt 2022). Mobile introns can move (or move a copy) from one genomic location to another, utilizing either an endonuclease or a reverse transcriptase. Introns may also move about the genome via several mechanisms (Yenerall and Zhou 2015), including transposition-like events or by entering at double strand break sites (DSBs). Repair of these insertions is an error-prone process that can result in direct repeats (DRs) surrounding the insert. Transposons can use similar processes to move within a genome, suggesting a common ancestry with introns (Park et al. 2022; Bendich and Rogers 2023). Introns and transposons also share other unifying characteristics, including modes of mechanisms of mobility and genes involved in mobility (for mobile members), and may be members of a large group of MGEs (Hickey and Benkel 1986; Yenerall and Zhou 2015; Hafez and Hausner 2015; Zimmerly and Semper 2015).

Here, we report that all types of introns examined have DRs at or near their borders and were likely inserted into DNA strand breaks by transposition-like events or via DNA repair systems, regardless of whether the breaks arise from an endonuclease or from events that are much less sequence-specific (e.g., radiation, oxidative, or glycation damage). The data indicate that introns have been inserted into regions of DSBs and might be part of a large single group of MGEs (which includes transposons) that moves in order to increase their numbers at the expense of the host, even if they occasionally and subsequently also benefit the host organism.

## Methods

Our main objective was to determine whether DRs were present at or near intron/exon borders for all four main types of introns among a broad range of taxa. We previously reported the presence of high densities of DRs in the rDNA intergenic spacers of a broad range of taxa (Bendich and Rogers 2023) and were curious as to whether DRs were also characteristic of intron integration. Nearly all intron publications report only sequences spanning or immediately adjacent to the intron/exon borders. The few publications describing DRs at the intron/exon borders are restricted to spliceosomal introns in a limited number of taxa (Roy 2004; Yenerall and Zhou 2015; Gozashti et al. 2022; Roy et al. 2023). We lengthened our searches to include sequences 40 bp upstream and 40 bp downstream (80 bp in total) of each of the intron/exon borders that could indicate integration events of MGEs, including non-identical repeats. We also broadened our searches to include all types of introns, DNA-containing organelles, and a broad range of taxonomic groups.

We began by selecting sequences from taxa that had been previously reported to contain introns (nuclear gene introns from *Homo sapiens* and mitochondrial gene sequences from *Schizosaccharomyces pombe*; see details below). The reason to start in this way was because of sequence availability. The majority (63%) of the 796,667 taxonomic nodes in the National Center for Biotechnology Information (NCBI) database (accessed on 14 August 2024) represent species of Metazoans and Fungi. Sequences were evaluated as to their annotations to assure accurate locations of the intron/exon borders. This included location of the GT-AG conserved border nucleotides, as well as the completeness of descriptions within the sequence database record. After evaluating the sequences from *Homo sapiens* and *S. pombe*, we selected sequences from additional species within Animalia [Metazoans] and Fungi, again using only sequences with accurate annotations of the intron/exon borders. This was followed by selection of plastid, mitochondrial, and nuclear introns within members of the Viridiplantae (Archaeplastida), and finally addition of introns from members of Bacteria, Archaea, Excavata, and Alveolata. Approximately 30% of the NCBI sequences represent Viridiplantae, 5% represent Bacteria, 0.2% represent Archaea, and the remaining 1–2% represent other taxa (including Alveolata, Amoebozoa, Eugleonozoa [Excavata], Rhizaria, and Stramenopiles). The species selected for this study (see below) reflect the taxonomic disparity in the national database.

Initially, we attempted to perform searches using bioinformatic, alignment, and sequencing tools to locate segments of similarity and/or identity (primarily DRs). However, none of these bioinformatic tools proved to be efficient and/or effective because we sought DRs of differing lengths and degrees of similarities/identities within two queried zones (the 5' and 3' splice regions), some of which were kilobases apart. Because our requirements were not satisfied by the available bioinformatics tools, we used an efficient and effective visual method that we had used previously to search for regions of similarity and complementarity within RNAs, including introns (e.g., Shinohara et al. 1996; Harris and Rogers 2011; Rogers 2019a). First, the 5' and 3' border regions (80 bp each) were placed vertically next to one another in a text file (with introns in red font and exons in black font). Then one of the sequences was shifted one base pair at a time until a region of similarity was reached. Where possible, the regions were extended until the longest region of sequence similarity was reached in the 5' and 3' intron/exon border sequences (i.e., DRs). Sequence similarities were calculated by presence or absence of identical nucleotides. Dissimilar bases, representing transitions or transversions were scored as positions only, gaps were excluded from the calculations. The longest DRs that were at least 70% similar were retained. One and two base pair non-identical bases (including gaps) were included if they linked two longer identical regions together. Although this manual inspection procedure was extremely laborious, it enabled us to examine introns from diverse prokaryotes and eukaryotes.

While the searches were limited to 80 bp surrounding the intron/exon borders, in all cases DRs were located much closer to each border: half the DRs were precisely at the borders or ± 2 bp from the borders; 80% were within 10 bp from the borders. The mean length of the DRs was 11.4 bp. The data were analyzed in several ways. First, the positions of the paired DRs in each sequence were categorized and tabulated according to whether the DRs were both at the exon/intron borders, within the introns, within the exons, or one in the intron and one in the exon. Second, the distribution of DR lengths was tabulated and plotted. Third, to evaluate the likelihood of chance findings, the probability of finding a sequence of bases within 80 bp of the downstream intron/exon border that matched a sequence of bases within 80 bp of the upstream intron/exon border was calculated for each DR length using STATA (StataCorp LLC, College Station, TX). The probability of a string of bp of length n to occur is 1/(4^n^). The null hypothesis is random (and equal) occurrence of A, G, C, or T in a given sequence. This is a well-defined formula throughout the scientific literature (Mount 2004). This null hypothesis was used for all taxa. Fourth, the distribution of distances from each DR to the exon/intron border was tabulated and plotted.

All sequences including introns were retrieved from the NCBI DNA sequence database during July through September 2022. Once an intron was selected for examination, it was never excluded from any of the analyses. Thus, all introns were evaluated based on their detailed annotation, representation of a broad phylogenetic range of Kingdoms and Phyla (including members of Alveolata, Animalia [Metazoa], Archaea, Bacteria, Eugleonozoa [Excavata], Fungi, and Viridiplantae [Archaeplastida]), organelle type (nucleus, mitochondrion, and plastid), and classification of intron type. The set included 2 group I and 2 group II introns from Bacteria and 4 archaeal introns from Archaea. Within Eukarya, group I introns were from the nuclei of species of Animalia (6) and Fungi (5); mitochondria of species of Animalia (1), Fungi (1), and Viridiplantae (1); and plastids of species of Viridiplantae (19); group II introns were from the nuclei of species of Fungi (1), mitochondria of Fungi (4), and Viridiplantae (30); and plastids of species of Eugleonozoa (28) and Viridiplantae (36); group III introns were from the plastids of Eugleonozoa (25); twintrons from plastids of species of Eugleonozoa (3); spliceosomal introns from the nuclei of species of Alveolata (15), Animalia (4), Eugleonozoa (1), Fungi (4), and Viridiplantae (21); and archaeal introns from the nucleus of a species of Fungi (1).

The sequences used were from the following: Alveolata [(*Symbidiodinium microadraticum* – dinoflagellate symbiont of jellyfish, nuclear spliceosomal introns, accession number LSRX01000131)]; Animalia [(*Amplexidiscus fenestrafer*, mitochondrial group II intron, accession number MH308002), (*Homo sapiens*, nuclear dystrophin gene spliceosomal introns, accession numbers AJ27220 and U60892), (*Ricordea yuma* – coral, mitochondrial group I intron, accession number MH308005), (*Carcharhinus leucas* – bull shark, nuclear rDNA ITS group I intron, accession number JN039366), (*Isurus oxyrhinchus* – shortfin mako shark, nuclear rDNA ITS group I intron, accession number MK079238), (*Stegobium paniceum* – bread beetle, nuclear rDNA group I introns, accession number D49657)]; Archaea [(*Methanospirillum hungatei,* tRNA archaeal intron, accession number NC_000916), (*Staphylothermus marinus*, rRNA archaeal intron, accession number NR_076485),* (Thermofilum pendens,* tRNA archaeal introns, accession number NZ_AASJ01000001)], Bacteria [(*Bacillus* sp. BSG40, bacterial group I intron, accession number AJ309312), (*Streptococcus agalactiae*, bacterial group II introns, accession numbers AF494487, AY189967), (*Thermotoga subterranea*, bacterial group I intron, accession number AJ556793)], Eugleonozoa [(uncultured tyrpanosome, nuclear spliced leader spliceosomal intron, accession number KR056281), (*Euglena gracilis*, plastid group II and group III introns, including some twintrons, accession number Z11874)], Fungi [(*Cenococcum geophyllum*, nuclear rDNA group I and group II introns, accession numbers Z48537, FJ013062), (*Mycoarachis inversa*, nuclear rDNA spliceosomal introns, accession number AB012953), (*Penicillium oblatum*, nuclear rDNA group I intron, accession number AB033529), (*Phialophora verrucosa*, nuclear rDNA group I intron), (*Saccharomyces cerevisiae*, nuclear spliceosomal and group I introns, accession number LBMA01000012), (*Saccharomycodes ludwigii*, nuclear archaeal intron, accession number NC_060204), (*Schizosaccharomyces pombe*, mitochondrial group II introns, accession number NC_001326), (*Scytalidium dimidatum*, nuclear rDNA group I intron, accession number AF258603), (*Xylaria polymorpha*, nuclear rDNA group I intron, accession number AB01404)], and Viridiplantae [(*Magnolia macrophylla* – bigleaf magnolia, plastid group I and group II introns, accession number AY687352), (*Triticum aestivum* – wheat, mitochondrial group II introns, accession numbers X75036, X57164; nuclear spliceosomal introns, accession number AJ512822; plastid group I and group II introns, accession number NC_002762), (*Vicia faba* – broad bean, mitochondrial group I and group II introns, accession number KC189947; nuclear spliceosomal introns, accession numbers AM886054, AJ277286; plastid group I and group II introns, accession number MT120813)].

## Results

**Fig. 2:**
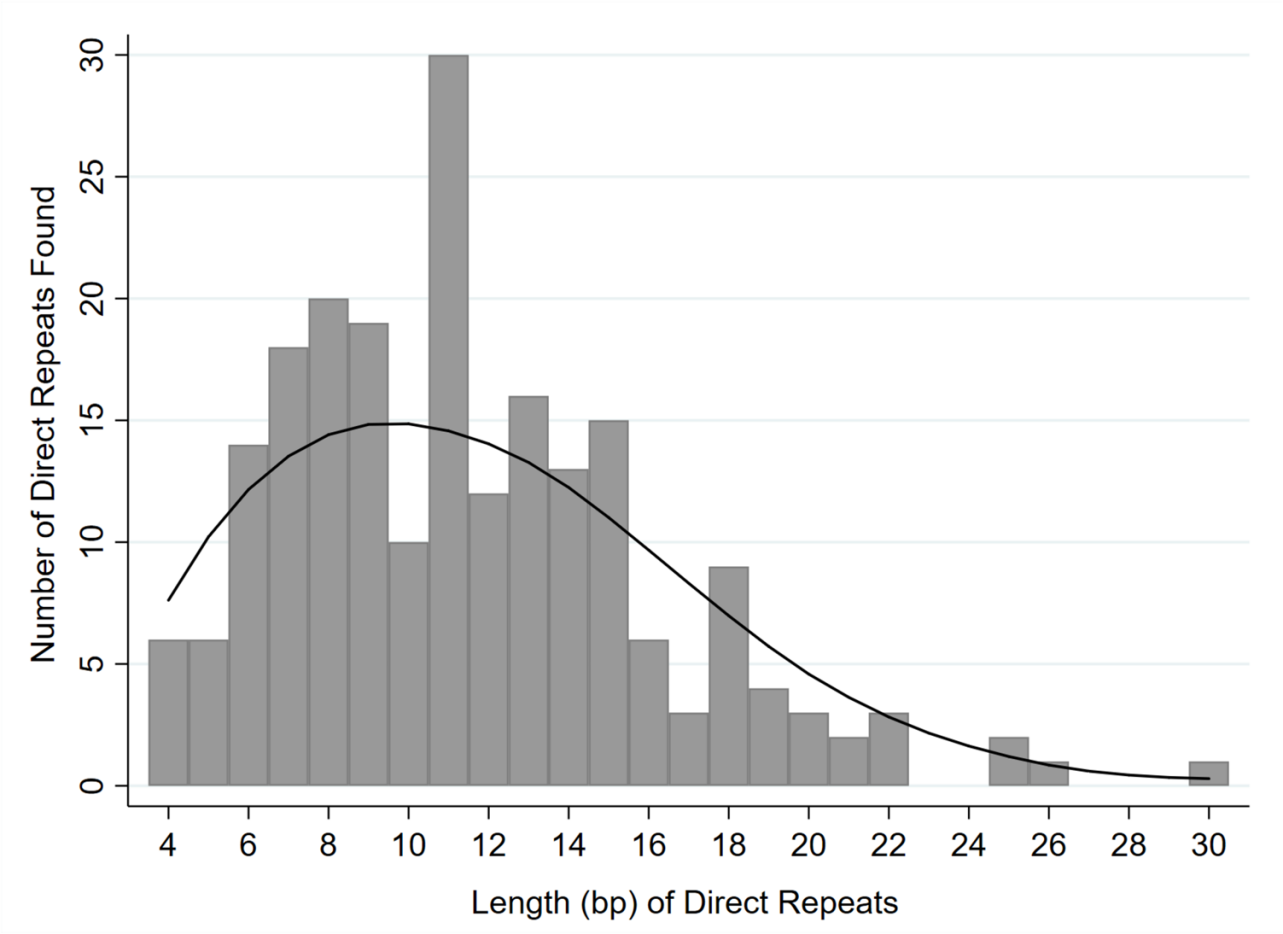
Distribution of DR lengths among the exon/intron borders. The most common DRs were 11 bp in length. The mean was 11.4. ± 4.6 bp. The line is the local weighted regression

DRs within a maximum of ± 40 bp from the 5' and 3' exon/intron borders (80 bp for each) were found in all of the 213 introns examined (without exception and without exclusion), including group I, group II, group III, spliceosomal, and archaeal introns, as well as group II and III twintrons (Table 1; Supplementary Table S1). Once we began to see possible patterns emerging, we added species, related and unrelated to those already analyzed. In every case (213 out of 213), a pattern emerged: regions at or surrounding the 5' intron/exon border contained a short DNA sequence that was directly repeated at or in the vicinity of the 3' intron/exon border. This pattern was, remarkably, shared by the intergenic sequence segment of eukaryotic ribosomal RNA that we attributed to the insertion (as well as removal, creating tandem direct repeats) of transposons (Bendich and Rogers 2023). The lengths of the repeated units ranged from 4 to 30 bp (Table 2; Fig. 2), with a mean of 11.4 bp (sd 4.6), and sequence similarities from 71–100%. In most cases the sequences of the 5' and 3' DRs differed, likely because the original insertion of the element occurred long ago (see e.g., Rogozin et al. 2012; Glick et al. 2024). The fact that they have maintained high similarities indicates possible positive selection to maintain the function of the surrounding gene sequences.

**Table 1:** Summary of matched direct repeats at or near the 5' and 3' exon/intron borders, including locations, frequencies, lengths, and distances relative to the exon/intron borders

Intron Type	Cell Location	Taxa Where Present^a^	5' exon	5' Repeat Location Frequency exon/intron border	intron	intron	3' Repeat Location Frequency intron/exon border	3' exon	Mean Repeat Length (bp)^b^	Mean Distance to e/i Border (bp)^c^
Group I	Nuclear	Al,Am,An,Fu, Pt,Rh, St	2/15	9/15	4/15	1/15	7/15	7/15	6.2 ± 3.4	0.0 ± 0.0 (0–0)
	Mitochondrial	Al,Am,An,Ex, Fu,Pt,St	1/2	0/2	1/2	0/2	0/2	2/2	8.0 ± 3.5	3.6 ± 5.3 (0–14)
	Plastid	Pt,St	4/20	4/20	12/20	7/20	4/20	9/20	9.9 ± 3.6	6.6. ± 7.1 (0–21)
	Prokaryote	Ba	2/2	0/2	0/2	1/2	1/2	0/2	16.0 ± 3.5	4.2 ± 4.2 (0–12)
	**TOTAL**	**––––-**	**9/39**	**13/39**	**17/39**	**9/39**	**12/39**	**18/39**	**8.8 ± 4.2**	**4.7 ± 6.6** **(0–21)**
Group II	Nuclear	Fu	0/1	1/1	0/1	0/1	1/1	0/1	10.5 ± 0.5	0.0 ± 0.0 (0–0)
	Mitochondrial	Al,An, Ex,Fu, Ha,Pt, Rh,St	9/33	10/33	14/33	15/33	8/33	10/33	12.0 ± 3.9	8.2 ± 9.0 (0–37)
	Plastid	Pt	19/66	17/66	30/66	24/66	20/66	22/66	11.4 ± 4.6	4.8 ± 6.4 (0–31)
	Prokaryote	Ar,Ba	1/2	1/2	0/2	0/2	1/2	1/2	10.8 ± 2.4	8.0 ± 8.0 (0–17)
	**TOTAL**	**–––––**	**29/102**	**29/102**	**44/102**	**39/102**	**30/102**	**33/102**	**11.6 ± 4.3**	**5.9 ± 7.6** (0–37)
Group III	Plastid	Ex	6/24	10/24	8/24	10/24	7/24	7/24	13.8 ± 5.1	5.0 ± 5.5 (0–22)
Spliceosomal	Nuclear	Al,Am,An,Ex, Fu,Pt, Rh,St	6/43	25/43	12/43	15/43	20/43	8/43	11.1 ± 5.1	3.6 ± 5.9 (0–29)
Archaeal	Nuclear	Al,An, Fu,Pt,St	1/1	0/1	0/1	0/1	0/1	1/1	5.5 ± 0.5	6.5 ± 6.5 (0–13)
	Prokaryote	Ar	2/4	2/4	0/4	1/4	2/4	1/4	10.9 ± 1.5	2.8 ± 2.4 (0–6)
	**TOTAL**	**–––––**	**3/5**	**2/5**	**0/5**	**1/5**	**2/5**	**2/5**	**9.8 ± 2.6**	**3.5 ± 3.9** **(0–13)**
	**Grand Totals**	**–––––**	**53/213** **(25%)**	**79/213** **(37%)**	**81/213** **(38%)**	**74/213** **(35%)**	**71/213** **(33%)**	**68/213** **(32%)**	**10.5 ± 2.4**	**4.4 ± 2.6**

^a^ Taxon names: *Al* = Alveolata; *Am* = Amoebae*; *An* = Animals; *Ar* = Archaea; *Ba* = Bacteria; *Ex* = Excavata; *Fu* = Fungi; *Ha* = Hacrobia*; *Pt* = Plants; *Rh* = Rhizaria*; *St* = Stramenopiles*. Presence of intron types from Rogers 2019a. *Introns present, but not sampled in this study

^b^ Mean and standard deviation are provided

^c^ Mean and standard deviation are provided. Range of lengths provided in parentheses

**Table 2:** Repeat Length Characteristics. Similarity of the direct repeats within each intron are presented, in addition to the number in each repeat length category, the mean similarities, and itemization of intron type

Repeat Length	Percent Similarity (Sequence Sources)^a^	Number	Mean Similarity	Gr I	Gr II	Gr III	Spl	Arch
4	100 (PII), 75 (NI), 75 (NI), 100 (PI), 80 (MII), 80 (Ar/Ar)	6	85 ± 11	3	2	0	0	1
5	80 (PI), 80 (Nsp), 80 (NI), 100 (Nsp), 100 (PI), 80 (NAr)	6	87 ± 9	3	0	0	2	1
6	100 (PII), 100 (PII), 100 (PIII), 100 (MII), 83 (NI), 100 (Nsp), 100 (MII), 83 (NI), 83 (Nsp), 100 (Nsp), 100 (PI), 83 (PII), 83 (PII), 83 (NAr)	14	93 ± 8	2	6	1	3	1
7	86 (Nsp), 86 (PI), 86 (PI), 71 (Nsp), 57 (Nsp), 86 (Nsp), 71 (Nsp), 71 (PII), 88 (PII), 71 (PII), 86 (PI), 86 (PII), 86 (PII), 71 (Nsp), 86 (MII), 86 (PII), 100 (NI), 100 (MII)	18	82 ± 11	4	8	0	5	0
8	88 (Nsp), 88 (PII), 88 (PII), 75 (PII), 88 (PII), 75 (NI), 75 (NI), 75 (NI), 86 (NI), 75 (PI), 75 (PII), 75 (PII), 75 (PII), 75 (Nsp), 75 (PII), 88 (Nsp), 75 (PI), 88 (PI)	18	80 ± 6	6	8	0	3	0
9	78 (BII), 78 (Nsp), 78 (PII), 78 (PII), 78 (PII), 78 (PII), 78 (PII), 89 (PIII), 89 (PIII), 89 (PIII), 89 (PIII), 100 (MII), 100 (PII), 89 (Nsp), 89 (PII), 78 (MII), 78 (NAr), 78 (Ar/Ar),	19	84 ± 7	0	10	4	2	2
10	70 (Nsp), 80 (Nsp), 80 (PI), 80 (PII), 80 (PII), 90 (PII), 90 (Nsp), 80 (PII), 80 (PII), 80 (Nsp)	10	81 ± 5	1	5	0	4	0
11	73 (MII), 82 (Nsp), 73 (Nsp), 82 (PII), 82 (PII), 73 (PII), 82 (PIII), 82 (PII), 82 (PIII), 82 (PIII), 82 (NI), 82 (NI), 73 (Nsp), 73 (PII), 82 (PIII), 100 (PIII), 82 (PII), 86 (PI), 86 (PII), 86 (PII), 71 (Nsp), 86 (MII), 86 (PII), 100 (NI), 100 (MII)	25	83 ± 8	3	12	6	4	0
12	75 (MII), 75 (MII), 75 (PII), 75 (PII), 83 (PIII), 83 (PII), 75 (MI), 75 (PII), 83 (PII), 83 (Nsp), 75 (MII), 75 (Ar/Ar)	12	78 ± 4	1	8	1	1	1
13	77 (BI), 70 (Nsp), 77 (Nsp), 77 (Nsp), 77 (Nsp), 77 (PI), 85 (PIII), 77 (PIII), 77 (PIII), 77 (PII), 77 (Nsp), 77 (Nsp), 85 (Nsp), 77 (MII), 77 (PII), 85 (Ar/Ar)	16	78 ± 4	2	3	3	7	1
14	71 (BII), 79 (Nsp), 71 (Nsp), 71 (Nsp), 79 (PII), 71 (PI), 71 (PII), 71 (PII), 79 (PIII), 79 (PIII), 79 (PII), 71 (Nsp), 86 (PI)	13	75 ± 5	2	5	2	4	0
15	73 (MII), 73 (MII), 73 (Nsp), 73 (Nsp), 73 (Nsp), 73 (PIII) 80 (PII), 87 (PII), 73 (PII), 87 (PII), 73 (PII), 80 (PIII), 73, (PII), 73 (Nsp), 73 (MII)	15	76 ± 5	0	9	2	4	0
16	81 (MII), 75 (Nsp), 75 (Nsp), 81 (MII), 81 (MII), 81 (MII)	6	79 ± 3	0	4	0	2	0
17	76 (PII), 76 (PIII), 71 (PI)	3	74 ± 2	1	1	1	0	0
18	72 (BI), 74 (BII), 78 (Nsp), 72 (Nsp), 78 (PII), 72 (MII), 72 (MII), 72 (MII), 78 (MII), 78 (MII)	10	75 ± 3	1	6	0	2	0
19	79 (PII), 84 (PII), 74 (PII), 79 (PI)	4	79 ± 4	1	3	0	0	0
20	80 (PII), 75 (PII), 75 (PII)	3	77 ± 2	0	3	0	0	0
21	81 (PII), 76 (MII)	2	78 ± 2	0	2	0	0	0
22	73 (PIII), 83 (PIII), 86 (PIII)	3	81 ± 6	0	0	3	0	0
23	–––––––	––	––	––	––	––	––	––
24	–––––––	––	––	––	––	––	––	––
25	84 (PII), 72 (PIII)	2	78 ± 6	0	1	1	0	0
26	73 (PII)	1	73	0	1	0	0	0
27	–––––––	––	––	––	––	––	––	––
28	–––––––	––	––	––	––	––	––	––
29	–––––––	––	––	––	––	––	––	––
30	73 (PIII)	1	73	0	0	1	0	0

^a^ Percent similarities were calculated by comparing the pair of direct repeats for each intron examined. Gaps were introduced to maximize paring of identical nucleotides. Abbreviations within the parentheses are as follows: *Ar* = archaeal, *B* = bacterial, *N* = nuclear, *M* = mitochondrial, *P* = plastid, *I* = group I, *II* = group II, *III* = group III, *sp* = spliceosomal

**Fig. 3:**
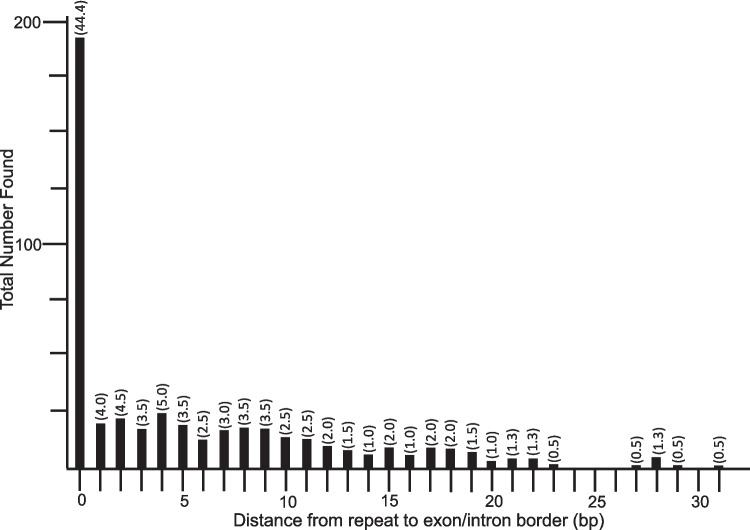
Distribution of the distances of the DRs from the exon/intron borders. The largest number of DRs (44.4%) were found either spanning the exon/intron borders or immediately adjacent to those borders, while 80% were within 10 bp of the borders

**Fig. 4:**
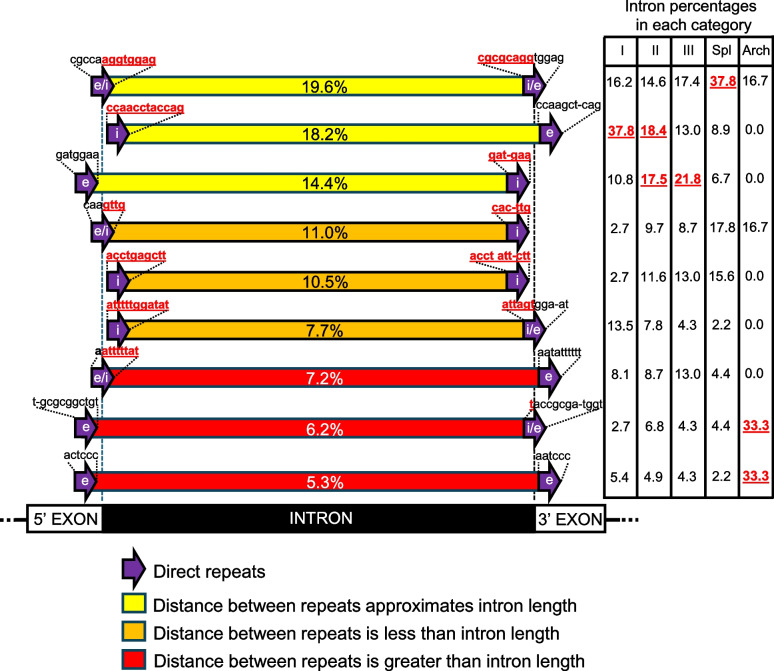
Frequencies of the positions of the DRs in the vicinity of the respective introns, arranged in decreasing frequencies. In the most frequent arrangement (19.6% of the cases, top bar), the 5' and 3' DRs spanned the exon/intron borders. The upper three bars indicate DRs separated by the length of the intron plus the several nucleotides added to create the DR (52.2% of the cases). The middle three bars indicate DRs separated by less than the length of the intron (29.2% of the cases); the lower three bars indicate DRs separated by more than the length of the intron (18.7% of the cases). DR on exon/intron border = e/i; DR in exon = e; DR in intron = i. Examples of 5' and 3' DR sequences are shown above each of the DR arrows. Sequences underlined in bold are within the introns. Other sequences are within the exons. A complete list of the DR sequences is provided in Supplementary Table S1. The table (right) shows the frequencies (as percentages) for each of the DR-pair categories for each type of intron (by column). The most common DR positions for each intron type are underlined in bold font

Probabilities of finding matching DRs in the upstream/downstream intron/exon borders are given in Table 3. Based on probability likelihoods, it was unlikely (*p* < 0.05) to find matching DRs when the lengths of the DRs were 6 or greater. It was more unlikely (*p* < 0.01) to find matching DRs when the DRs were 7 or greater. The distances to the exon/intron borders ranged from 0 (spanning the border site) to 31 bp (Fig. 3), with a mean of 5.0 bp (sd 6.6), with 44% spanning, or immediately adjacent to, the exon/intron borders. More than 80% were within 10 bp of the intron/exon borders. Therefore, the search areas for all DRs were less than the maximums (80 bp each) for each pair of DRs. While there were slightly fewer DRs (26%) located in the 5' exons (Fig. 4; Table 1), the percentages for all other locations were close to the same values (33–37%). DRs on the 5' exon/intron border were most often paired with DRs on the 3' intron/exon border (19.6% of all introns examined; Fig. 4, upper bar). DRs near the 5' end of the intron were most often paired with DRs in the 3' exon (18.2%; Fig. 4, second bar), while DRs in the 5' exon were most often paired with DRs near the 3' end of the intron (14.4%; Fig. 4, third bar). All three of these arrangements approximated the total lengths of the respective introns (total of 52.2%; Fig. 4, upper three bars), the likely length of the original insert. All other combinations of DRs were either shorter or longer than the respective introns (Fig. 4, middle three and lower three bars, respectively). Each of the intron types exhibited a variety of DR arrangements (Fig. 4, table on right). The most common DR locations for spliceosomal introns (37.8%) were at the exon/intron borders; and the most common locations for group I, II, and III introns included one DR within the intron and the other DR in one of the exons, without altering the length of the intron (Fig. 4, upper three bars). Each of these arrangements maintains the lengths of the mature RNAs after splicing, but two of the three change their end sequences. Intron versions that decrease (middle three bars) and increase (lower three bars) the lengths of the splice products also were found. Although fewer archaeal introns were analyzed, most would alter the length of the mature RNA.

**Table 3:** Probabilities of a sequence within 80 bp of the 5' intron/exon border being present within 80 bp of the 3' intron/exon border, indicated by length of direct repeat (DR)

Length of DR	Number of DRs Found	Possible Combinations of Nucleotides in DR	Probability of Finding a Random DR Match
4	6	256	0.300781250
5	6	1,024	0.074218750
6	14	4,096	0.018310547
7	18	16,384	0.004516602
8	20	65,536	0.001113892
9	19	262,144	0.000274658
10	10	1,048,576	0.000067711
11	30	4,194,304	0.000016689
12	12	16,777,216	0.000004113
13	16	67,108,864	0.000001013
14	13	2.684354560E + 08	0.000000250
15	15	1.073741824E + 09	0.000000061
16	6	4.294967296E + 09	0.000000015
17	3	1.717986918E + 10	0.000000004
18	9	6.871947674E + 10	0.000000001
19	4	2.748779069E + 11	< 0.000000001
20	3	1.099511628E + 12	< 0.000000001
21	2	4.398046511E + 12	< 0.000000001
22	3	1.759218604E + 13	< 0.000000001
23	0	7.036874418E + 13	< 0.000000001
24	0	2.814749767E + 14	< 0.000000001
25	2	1.125899907E + 15	< 0.000000001
26	1	4.503599627E + 15	< 0.000000001
27	0	1.801439851E + 16	< 0.000000001
28	0	7.205759404E + 16	< 0.000000001
29	0	2.882303762E + 17	< 0.000000001
30	1	1.152921505E + 18	< 0.000000001

In summary, each of the introns analyzed was bounded by a pair of DRs (4–30 bp). For all introns analyzed, the locations of some of the DRs indicated that a spliced RNA transcript would rectify the gene defect caused by intron insertion. For each category of intron, however, most DR locations would alter the length and/or end sequences of the mature RNA leading to an altered gene product.

## Discussion

We previously reported high concentrations of DRs in the rDNA intergenic spacers of a taxonomically-diverse set of organisms (Bendich and Rogers 2023). These DRs are indicative of transposition insertions and/or DSB-repair mechanisms (Park et al. 2022; Yenerall and Zhou 2015). Based on our research on introns (e.g., Rogers 2019a), we wondered whether introns also originated from such mechanisms and began a search for DRs in the vicinity of the 5' and 3' splice sites, which had been reported sporadically for a limited number of spliceosomal introns (Gozashti et al. 2022; Roy 2004; Roy et al. 2023; Yenerall and Zhou 2015). Here, we sought to broaden the searches by examining all types of introns (group I, group II, group III, twintrons, spliceosomal, and archaeal) in both closely and distantly related species. Initially, we used available online tools to search for DRs, sequence similarities, and sequence alignments. Each tool was inefficient and unable to readily identify short repeats that we subsequently found by visual inspection. Inefficiency was due to the need to first locate accurately-annotated sequences that included introns and then to compare the region surrounding the 5' intron/exon border with the region surrounding the corresponding 3' intron/exon border, which were sometimes separated by kilobases of intervening sequence and often were imperfect repeats. Many of the repeats exhibited mutations, including transitions, transversions, and indels. Such mutations were not surprising because many intron insertions were present more than 1.5 billion years ago in the last eukaryotic common ancestor (Rogozin et al. 2012; Glick et al. 2024). We then switched to manual searching that we had used previously to study introns (Harris and Rogers 2011; Rogers 2019a; Shinohara et al. 1996). Though this method was tedious, it yielded the results presented in this paper (see Supplementary Table S1): DRs ranging in length from 4 to 30 bp (DRs 3 bp and shorter were not included in our data because of statistical considerations). Here, we report DRs at or adjacent to the borders of all 213 introns examined in seven major taxa of Bacteria, Archaea, and Eukarya (including those in nuclei, mitochondria, and plastids; Figs. 2, 3 and 4, Tables 1, 2 and 3; Supplementary Table S1).

**Fig. 5:**
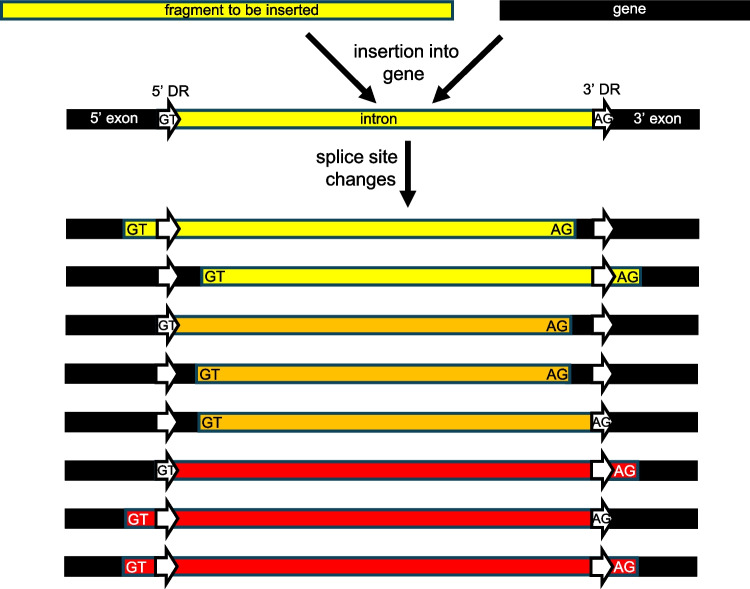
Model for intron insertion and splice site locations. A DNA fragment enters a genic break site caused by either transposon integration, an endonuclease, or a nonspecific act of damage. Error-prone repair of the break produces direct repeats at the 5’ and 3’ borders of the insert. Recombination may also lead to an insert flanked by DRs. Initially, the consensus 5'GT and 3'AG would be used as the splice sites (shown inside the DR arrows). However, following mutations of the original GT and/or AG sequences, cryptic splice sites (also GT and/or AG sequences) may have already existed or been produced by subsequent mutations, which can lead to changes in the positions of splice sites (shown within the original exon and/or intron regions). The resulting splicing patterns may lead to mature RNAs that reflect the original intron length (upper three bars), shorter RNAs (middle three bars), or longer RNAs (lower three bars), as reported in Fig. 4. The predominant GT and AG splice sites are shown in each case

The presence of these DRs suggests that transposition-like and/or DSB-based insertion events occur by similar mechanisms among diverse organisms, even though introns are sparse in prokaryotes but numerous in most eukaryotic genes. For both introns and transposons, mobile element insertion typically involves DNA strand breaks (at either specific or random sites), single-stranded regions at staggered ends, and damage repair synthesis using error-prone polymerases (Hedges and Deinenger 2007; Muñoz-López and García-Pérez 2010; Bendich and Rogers 2023). The DRs in the present study ranged from 4 to 30 bp, with a mean of 11.4 bp, more than half of which were at the intron/exon border ± 2 bp, and 80% of which were within 10 bp of the intron/exon borders (Fig. 3). That DRs were present for 100% of the introns chosen for analysis before their ends were examined and that DRs were found within mRNA, tRNA, and rRNA genes, indicates they were likely inserted into DSBs by transposition-like or analogous events. This is yet another characteristic that unites introns and transposons, many of which may be within a single group of MGEs. The fact that the DRs are not always precisely at the splice sites (Fig. 4) suggests that mobile element insertion is followed by evolutionary selection for the most successful splice sites that produce advantageous products (Fig. 5). Cryptic splice sites may become the most often used sequences, potentially increasing or decreasing the length of the mature RNA or producing more than one version of mature RNA.

Similarities between introns and transposons have long been recognized (Hickey and Benkel 1986; Roy 2004). Reverse transcriptases used by retrotransposons and group II introns (and retroviruses) have similar properties (Nakamura et al. 1997; Aizawa et al. 2003; Roy 2004; Rogozin et al. 2012; Monachello et al. 2021; Roy et al. 2023). Endonucleases within mobile introns have similarities to transposases in DNA transposons (Perotto et al. 2000; Bonocara and Shub 2009; Nawrocki et al. 2018; Rogers 2019a). Our proposed mechanism for intron insertion is analogous to transposition or mechanisms involving repair of DSBs, the major difference being the ability to accurately splice exons to maintain gene functions among introns. The arriving DNA or RNA element may insert in a location that already includes borders compatible with splicing or the element may have a sequence resembling an exon/intron border (Hallsten et al. 2011), while the other border already exists in the flanking sequences (Fig. 5). While most insertions will be detrimental, occasionally a beneficial intron may emerge. Some introns become intertwined, creating twintrons (Hafez and Hausner 2015), and introns themselves are hotspots for additional transposon or intron integration (Marchant et al. 2022). In addition to mobility, introns can exhibit plasticity. One group I intron near the 3' end of the SSU rRNA gene in fungi folds in both a group I as well as a group II form, with intron borders shifted by only one nucleotide on one end and two nucleotides on the other end (Rogers et al. 1993; Shinohara et al. 1996; Harris and Rogers 2011; Rogers 2019a). This intron might be an example of interconversion between a group II and group I intron, linking them in an evolutionary sense. In another case, several species of fungi contain group I introns within the SSU rDNA that range from 62–78 nucleotides, which are missing most of the canonical central portions of the introns, thus potentially causing non-functional SSU rRNAs. However, parts of the adjacent 3' SSU and ITS1 (internal transcribed spacer 1) of the rDNA compensate for the missing sections to allow accurate splicing of the SSU rRNA (Harris and Rogers 2008, 2011; Simmons et al. 2015; Rogers 2019a). These findings indicate that exon/intron lengths and borders can change substantially to allow recovery of functional RNAs.

Whereas the upper three bars in Figs. 4 and 5 indicate that intron-length inserts comprise most of all inserts analyzed, the fraction of all introns that carry DRs at both borders of the original intron was highest for spliceosomal introns (37.8%; Table in Fig. 4) among the five intron categories. This relatively low flexibility in DR placement probably reflects the need to maintain the translational reading frame to produce a functional protein, whereas for the other four intron categories splicing produces mature transcripts less likely to be functionally compromised because they are not subjected to the rigors of translation. In all cases, however, most of the transcripts do not represent a “clean” removal of intronic RNA to rescue the product of the original gene.

Most DR arrangements for group I introns included one copy of the repeat unit near the 5' end of the intron and the other copy near the 5' end of the 3' exon, without altering intron length (Figs. 4 and 5, upper three bars). However, the location of the DRs indicates a shift in intron/exon borders after the initial integration. This entails mutation of one or both of the splice sites (i.e., the GT and AG consensus sequences) and subsequent mutations elsewhere to create an alternative GT and/or AG site. Group I introns were almost exclusively found in rRNA and tRNA genes. Because there are no constraints to maintain a reading frame for these RNAs, selection is primarily based on whether the mature RNAs can fold into their functional conformations, which are primarily dependent on the lengths of the RNA segments rather than specific nucleotides. The DR pairs flanking group II and III introns (found primarily in mitochondria, plastids, and prokaryotes) are less positionally constrained than either group I or spliceosomal introns, but the distance between DRs still approximates the intron lengths. Most archaeal introns examined had DR distances that would alter one or both exons (Fig. 4). Because most introns in tRNA genes are in the anticodon loop segment, some variability in the mature tRNAs and their anticodons should result. The evolution of tRNAs and their cognate aminoacyl tRNA transferases (aaRSs) is complex, including some aaRSs that charge biochemically-similar tRNAs and modify tRNAs leading to a diverse set of tRNAs (Berget et al. 1977; Rogers 2019b). While this may result in faulty translation, proofreading enzymes adjoining the aaRSs can replace the incorrect amino acid with the correct amino acid (Rogers 2019b). Additionally, posttranslational modification systems exist that can alter the amino acid sequence in the final protein. Changes in tRNA specificity caused by intron insertion may, therefore, be tolerated or even occasionally beneficial during evolution, creating additional tRNA diversity.

In 29.2% of the cases the distance between the DRs was shorter than the intron (Figs. 4 and 5, middle three bars), creating an extension of one or both exons and mature RNAs that would be longer than the ancestral version before intron insertion. Conversely, in 18.7% of the cases (Figs. 4 and 5, lower three bars) the distance between the DRs exceeded the intron length, which would shorten the mature RNA. Lengthened and shortened RNAs should negatively affect the resulting gene products, illustrating the deleterious effects of most introns on the cell. Some changes may result in additional beneficial gene versions. When the host cell occasionally survives an intron insertion, however, the intron itself is the primary beneficiary of the insertion event, with secondary benefits accruing to the host.

Why are genes in pieces? This question has existed since introns were discovered (Berget et al. 1977; Chow et al. 1977; Gilbert 1978). Most proposed answers emphasize the benefits to organisms carrying introns, such as expanding the proteome and increased flexibility of gene expression. Although these organismal benefits are unquestioned, we suggest that the original beneficiary was the intron itself acting as a selfish MGE, with organismal benefits appearing later in evolution. Damage to DNA in all genomes is unavoidable. Repair of the most severe type of damage, a double strand break (DSB), must be successful if the cell is to survive. We previously proposed that the insertion of a transposon involves a DSB requiring repair (Bendich and Rogers 2023), and we now propose the same for insertion of an intron. In bacteria, the fraction of the genome occupied by transposons plus transposon-defense genes is only about one or two percent (Koonin and Makarova 2017; Gao et al. 2020; Kirchberger et al. 2020) and an even smaller fraction is attributed to introns. The fraction in eukaryotic nuclei is larger, the genomes are larger, and the number of introns is in the thousands and tens of thousands (Sakharkar et al. 2004). In many eukaryotes the number of nucleotides in introns of a gene is greater than that in the exons, and splicing errors are common (Xu and Zhang 2021). The major unanswered question is: what accounts for the permissive nature of eukaryotic genomes that has allowed the dramatic rise of introns, transposons, and other MGEs?

## Supplementary Information

Below is the link to the electronic supplementary material.

Supplementary file1 (PDF 382 KB)
