# Supplementary material for: Direct repeats found in the vicinity of intron splice sites
Source: Naturwissenschaften. 2025 Jan 30;112(1):14. doi: 10.1007/s00114-025-01966-4 (PMC11782384; doi:10.1007/s00114-025-01966-4)
Supplement: Supplementary file 1 — Supplementary file1 (PDF 382 KB) [file 114_2025_1966_MOESM1_ESM.pdf]

Supplementary Table S1. Direct Repeats at or adjacent to the 5' and 3' exon/intron borders.

Taxon	Genome[a]	Gene	Type[b]	5' Border[c]	3' Border[c]	5' Repeat[d] (distance to e/i border)	3' Repeat[d] (distance to i/e border)	DR Lengths (nt)[e]	Search Length (nt)[f]	Accession
**ANIMALIA**										
Amplexidiscus fenestrafer	Mt	coxI	II	i	e	tg-a (0)	tgga (0)	3/4	7	MH308002
Homo sapiens	Nu	dystrophin	Spl (7)	i	i	tttt-g (7)	ttttag (0)	5/6	18	U60822
	Nu	dystrophin	Spl (8)	e/i	e	gaaagttggta (0)	ga-tgttgata (0)	11/10	21	"
	Nu	dystrophin	Spl (9)	e/i	i/e	ttctcaacaggtaa (0)	ttctctgcagatca (0)	14/14	28	"
	Nu	dystrophin	Spl (49)	e	i/e	ctcagccagt (4)	ctaagccagt (0)	10/10	20	AJ271220
Ricordea yuma	Mt	cox1	I	i	i/e	atttttggatat (14)	attagtgga-at (0)	12/11	24	MH308005
Carcharhinus leucas	Nu	rDNA ITS	I	e/i	i/e	tctgctgg (0)	tctga-gg (0)	8/7	15	JN039366
Isurus oxyrinchus	Nu	rDNA ITS	I	i	e	gtctg (0)	gcctg (2)	4/4	12	MK079238
Stegobium paniceum	Nu	rDNA	I (1)	e/i	i/e	cgg-att (0)	cggcatt (0)	6/7	13	D49657
	Nu	rDNA	I (2)	e	e	aacggg (1)	aacggg (1)	6/6	14	"
	Nu	rDNA	I (3)	e	e	gg--tgg (2)	ggcatgg (0)	5/7	14	"
	Nu	rDNA	I (4)	e/i	e	ttgat (0)	tagat (0)	5/5	10	"
	Nu	rDNA	I (5)	e/i	i/e	acaaggtt (0)	aca-cgtt (0)	8/7	15	"
**ALVEOLATA**										
Symbiodinium microadriaticum	Nu	hypothetical	Spl (1)	i	e	gtccg (0)	gtccg (7)	5/5	17	LSRX01000131
	Nu	"	Spl (2)	e/i	i	caagttg (0)	cac-ttg (4)	7/6	17	"
	Nu	"	Spl (3)	e/i	i	tgaatcggaccct (0)	tgcct-ggacc-t (4)	13/11	28	"
	Nu	"	Spl (4)	e/i	i/e	aacttggacagg (0)	aactttgtgcagg (0)	12/12	24	"
	Nu	"	Spl (5)	e	e	gcagga (9)	gcagga (4)	6/6	25	"
	Nu	"	Spl (6)	e/i	i/e	agcgcttgtc (0)	ag-gcttt-tc (0)	10/8	18	"
	Nu	"	Spl (7)	e/i	i/e	aggag (0)	aggag (0)	5/5	10	"
	Nu	"	Spl (8)	i	ie	ccaaaatc (0)	ccaaggtc (0)	8/8	16	"
	Nu	"	Spl (9)	i	e	gctgcca-gact (11)	gctgcaacgact (9)	11/12	43	"
	Nu	"	Spl (10)	e/i	i/e	ggcgagga (0)	ggc-agga (0)	8/7	15	"
	Nu	"	Spl (11)	e/i	e	accttgggt-ttcgtc (0)	acatgggtgttcctc (2)	14/15	31	"
	Nu	"	Spl (12)	e/i	i/e	tggatgtaccccgcc (0)	tgcaggtat-ccgcc (0)	15/14	29	"
	Nu	"	Spl (13)	e/i	i/e	cgccaaggtggag (0)	cgcgcaggtggag (0)	13/13	26	"
	Nu	"	Spl (14)	e/i	i/e	tcaga-cagga (0)	tctgaagagga (0)	10/11	21	"
	Nu	"	Spl (15)	e/i	e	aggctgggg (0)	aggc-gggg (1)	9/8	18	"
**FUNGI**										
Phialophora verrucosa	Nu	rDNA (1514)	I	e/i	i/e	taggtga (0)	taggtga (0)	7/7	14	X65199
Penicillium oblatum	Nu	rDNA (S788)	I	i	e	caagat (0)	ca-gat (0)	6/5	11	AB033529
Cenococcum geophyllum	Nu	rDNA (S1514)	I	e/i	i/e	aaggtttccgt (0)	aacgt-tccgt (0)	11/10	21	FJ013062, Z48537
"	Nu	rDNA (S1512)	II	e/i	i/e	aaggtttccgt (0)	aacgt-tccgt (0)	11/10	21	"
Xylaria polymorpha	Nu	rDNA (S943)	I	e/i	e	caag (0)	ca-g (0)	4/3	7	AB014043
Saccharomycodes ludwigii	Nu	tRNA-G	Arc	e	e	actccc (6)	aatccc (19)	6/6	37	NC_060204
Mycoarachis inversa	Nu	rDNA CI-1	I	e/i	i	tgaacg (0)	t-aacg (0)	6/5	11	AB012953
"	Nu	rDNA CI-2	I	e/i	e	taaaacg (0)	taaa-cg (0)	7/6	13	"
Saccharomyces cerevisiae	Nu	RPL8B	Spl	e/i	i/e	a-gtatg (0)	aggt-tg (0)	6/6	12	LBMA01000012

"	Nu	ADH2	Spl	e/i	i/e	agtatgt (0)	agg--gt (0)	7/5	12	"
"	Mt	cox I αI5B	I	i	e	a-tta (2)	actta (0)	4/5	9	"
Schizosaccharomyces pombe	Mt	coxI	II	e/i	i	ggtggtggt (0)	ggtggtggt (5)	9/9	23	NC_001326
"	Mt	coxI	II	i	i	aattta (2)	aattta (2)	6/6	16	"
"	Mt	cob	II	e	i/e	attatctgataaaat (0)	atgatttgataacaat (0)	16/16	32	"
Scytalidium dimidatum	Nu	rDNA	I	i	i/e	tggcctgt (0)	ttgc-tgt (0)	8/7	15	AF258603
**ARCHAEA**										
Methanospirillum hungatei	Arc	tRNA	Arc	e	i/e	t-gcgcggctgt (6)	taccgcga-tgt (0)	11/11	24	NC_000916
Staphylothermus marinus	Arc	rDNA	Arc	e/i	i	aacgggaatcccg (0)	aacggga-ccccg (5)	13/12	30	NR_076485
Thermofilum pendens	Arc	tRNA	Arc	i	e	cgc-c (0)	cgctc (6)	4/5	15	NZ_AASJ01000001
"	Arc	tRNA	Arc	e/i	e	ccgggg-cg (0)	ccgggttcg (5)	8/9	22	"
**BACTERIA**										
Bacillus sp. BSG40	Bac	bnrdF	I	e	i	tttaattatc-cg (12)	tttaagtatagcg (20)	12/13	57	AJ309312
Streptococcus agalactiae	Bac	lplA-1	II	e	e	ttttg-tat (17)	ttttgattt (15)	8/9	49	AF494487
"	Bac	ftsY	II	e/i	i/e	tttat-agt-acga (0)	tttaaaagacacga (0)	12/14	26	AY189967
Thermotoga subterranea	Bac	Tsu.bL1917	I	e	i/e	taaacg-gcgg-ccgtaac (4)	taa-cgaacggtcc-taag (0)	17/17	36	AJ556793
**ARCHAEPLASTIDA**										
Magnolia macrophylla	Pl	rps12	II	i	i	acctagagctt (6)	acctatt-ctt (12)	11/10	39	AY687352
"	Pl	trnK	I	i	e	ctcat (3)	ctcat (8)	5/5	21	"
"	Pl	rps16	II	e/i	e	ctaccga (0)	c-aacga (2)	7/6	15	"
"	Pl	trnG	I	i	i/e	agcaagataattgttga (5)	agc-tgata-ttggggga (0)	17/15	37	"
"	Pl	atpF	II	e/i	i	aattactttcat (0)	aagaacttt-at (5)	12/11	28	"
"	Pl	rpoC1	II	e/i	i	tatccaaagcta (0)	tat-taaagcta (9)	12/11	32	"
"	Pl	ycf3	II	i	i/e	cct-tatc (3)	cctctatc (0)	7/8	18	"
"	pl	ycf3	II	e/i	i/e	gc-t-gtag-g (0)	gcgtggtagag (0)	8/11	19	
"	Pl	trnL	I	i	e	ct-aac-ct (0)	ctgaaatct (9)	7/9	25	"
"	Pl	trnV	I	e/i	i/e	gc-ca-ttttg (0)	gcgcacatttg (0)	9/11	20	"
"	Pl	rpl12	II	i	e	gacttag (0)	ga--tag (0)	7/5	12	"
"	Pl	clpP	II	e/i	i/e	ttgggact (0)	ttcgcact (0)	8/8	16	"
"	Pl	clpP	II	i	e	gttttaacataggaa (8)	gtttg-acgtag-aa (10)	15/13	46	"
"	Pl	petB	II	i	e	atattaacta (11)	atat--acta (5)	10/8	34	"
"	Pl	rpl2	II	i	e	tt-catc (2)	ttccatc (4)	6/7	19	"
"	Pl	ndhB	II	i	i	cagcttttct (0)	cagcttg-ct (7)	10/9	26	"
"	Pl	trnI	I	e	i	cta-ttaa (2)	ctact-aa (8)	7/7	24	"
"	Pl	trnA	I	i	e	ccaacctacag (19)	ccaagct-cag (13)	11/10	53	"
"	Pl	ndhA	II	e/i	i/e	ttagtat-cagctactat (0)	ttagtgtgcatct-ctat (0)	17/17	34	"
"	Pl	trnA	I	e	i	ctgtaggc-gg (0)	ctgtaggttgg (19)	10/11	40	"
"	Pl	trnI	I	i	e	ttagtag (8)	ttaatag (3)	7/7	25	"
"	Pl	ndhB	II	e	e	gaggactg (12)	gaggg-tg (1)	8/7	28	"
"	Pl	rpl2	II	e	i	gatggaa (4)	gat-gaa (2)	7/6	19	"
Triticum aestivum	Mt	nad7 (1)	II	i	i	g-tgcggcacg (0)	gatccggc-cg (17)	10/10	37	X75036
"	Mt	nad7 (2)	II	e	i/e	tttaccttattt-tg (10)	ttgaccctatctatg (0)	14/15	39	"
"	Mt	nad7 (3)	II	i	i/e	caatatggggga (19)	caacctggggta (0)	12/12	43	"
"	Mt	nad7 (4)	II	e	i/e	ctatccatggaat-c (0)	cta-cc-t-caattc (0)	14/11	25	"

"	Mt	nad4 (1)	II	e	i	gtgccaatccct (5)	gggcca-ccccct (8)	12/11	36	X57164
"	Mt	nad4 (2)	II	i	i	ggcggcgtat--gg-tc (4)	gggggaatattaggctc (3)	14/17	38	"
"	Mt	nad4 (3)	II	e/i	i/e	ttcttgttggaggggg (0)	tacttgttcggatggg (0)	16/16	32	"
"	Nu	stD1.1a (1)	Spl	i	i	ttctttcc (7)	ttatttcc (1)	8/8	24	AJ512822
"	Nu	stD1.1a (2)	Spl	e/i	i/e	tacctcaggtaacatc (0)	tacgccagg-a-catc (0)	16/14	30	"
"	Nu	stD1.1a (3)	Spl	i	i	attaccttttttca (18)	attttcttttgca (0)	13/13	26	"
"	Nu	stD1.1a (4)	Spl	e	i/e	ct-caggcgatgctt (10)	ctgcaggctagggtt (0)	14/15	39	"
"	Nu	stD1.1a (5)	Spl	i	e	caacctt-ttgtctattt (12)	catccttcttggcg-ttt (29)	17/17	75	"
"	Nu	stD1.1a (6)	Spl	e	i	ccttct-tggcgt (23)	ccttctgtgt-gt (21)	12/12	68	"
"	Nu	stD1.1a (7)	Spl	e	i	gttg--caatt (0)	gttgatcaatt (15)	9/11	35	"
"	Nu	stD1.1a (8)	Spl	e	e	aaacgtcatta (7)	aaactttatca (1)	11/11	28	"
"	Nu	stD1.1a (9)	Spl	i	i	actacaaatttagt (3)	actgacaagatatgt (9)	14/14	42	"
"	Nu	stD1.1a (10)	Spl	e/i	i	ttgcggtgcgaacta (0)	ttgca-tg-gaa-ta (2)	15/12	27	"
"	Nu	stD1.1a (11)	Spl	i	i/e	cttcttttctacaa (5)	ctatttttcagaa (0)	13/13	33	"
"	Nu	stD1.1a (12)	Spl	e/i	i	ctatctcgtaa (0)	ctgtctactaa (11)	11/11	33	"
"	Nu	stD1.1a (13)	Spl	e/i	e	agg-taaaatacca (0)	agaacaaaat-cca (16)	13/13	42	"
"	Pl	psbK	II	i	e	atttg-tt (17)	atttgctt (22)	7/8	54	NC_002762
"	Pl	trnI	I	e	i	ag-caat (16)	agtcaat (18)	7/8	47	"
"	Pl	atpF	II	i	i/e	agat-aaagttcttatctaa (10)	agatgaaagtaattttctaa (0)	19/20	49	"
"	Pl	ycf3 (1)	II	i	i	cagcct-tatc (0)	ca-cctctatc (6)	10/10	26	"
"	Pl	ycf (2)	II	e	i/e	cggttgtag (1)	cg-tggtag (0)	9/8	18	"
"	Pl	trnL	I	i	i/e	atggaaa (18)	aaggaaa (0)	7/7	32	"
"	Pl	trnV	I	e/i	i/e	cg-gtaaaac-ag (0)	cgtgtaaa-cgag (0)	11/12	23	"
"	Pl	petB	II	e/i	e	gaatatgagtgtgt (0)	gtatatgattg-gt (3)	14/13	53	"
"	Pl	petD	II	i	e	gaatta-ttgat (9)	gacttaaatgat (12)	11/12	44	"
"	Pl	rpl16	II	i	e	tatc (3)	tatc (7)	4/4	18	"
"	Pl	rpl2	II	i	e	tccatc (3)	tccatc (4)	6/6	19	"
"	Pl	ndh2	II	e/i	i	catccctcagtctcag-cttt (0)	cat-cctc-gt-tcagtcttt (28)	20/17	66	"
"	Pl	ndh1	II	e/i	e	atg-taacaaggat-ataga (0)	atgataataa-cataataga (2)	18/19	39	"
"	Pl	ndhA	II	i	i/e	tagtatcagctactattg-tagtgt (0)	t-gcatct-cta-taacgatcgtgt (0)	24/21	46	"
"	Pl	trnA	I	i	e	tggt-ggc-ggaga (8)	tggttgactcgata (18)	12/14	52	"
"	Pl	trnI	I	i	i	ggacc-tgtt (20)	ggtccgtgtt (9)	9/10	48	"
"	Pl	ndh2	II	i	i/e	aattcctacctcta (16)	actccctac-tcca (0)	14/13	43	"
"	Pl	rpl2	II	e	i	ctacct (4)	ctacct (3)	6/6	19	"
Vicia faba	Nu	cvc	Spl (1)	e/i	e	agcggtaatt (0)	agcgata-tt (4)	10/9	30	AM886054
"	Nu	cvc	Spl (2)	e/i	e	agatcttga-ggt (0)	a-atctttatcgt (11)	12/12	35	"
"	Nu	cvc	Spl (3)	i	e	agcaacata (11)	ag-accata (10)	9/8	38	"
"	Nu	cvc	Spl (4)	e	i/e	tttct-cggagattt (5)	tttatagggagttt (0)	13/14	32	"
"	Nu	cvc	Spl (5)	e	i	gaaacttt (7)	gaaaattt (4)	8/8	27	"
"	Nu	enod18	Spl (1)	i	i	ttctatattttta (5)	ttcttcattttgta (2)	14/14	35	AJ277286
"	Nu	enod18	Spl (2)	e/i	i/e	g-aggtg (0)	gtaggtg (0)	6/7	13	"
"	Nu	enod18	Spl (3)	e/i	i/e	aatcaaaagg (0)	aatt-atagg (0)	10/9	19	"
"	Mt	nad2	II	e/i	i	acttagtgtttc-gcgcc (0)	acgt-gtggttctg-gcc (17)	17/16	50	KC189947
"	Mt	nad5	II	e/i	i/e	cttctttggcgaccgtg (0)	cttct--gactccgtg (0)	17/15	32	"
"	Mt	rps3	II	i	i	accgcgaaagtcaccgaatg (5)	acggcg-gagtcacc-attg (6)	20/18	49	"
"	Mt	nad7	II	i	i	gctactagga--catcagt (11)	gctactag-atgcaa-agt (10)	17/17	55	"
"	Mt	nad7	II	i	e	gtggtga (17)	gtggtga (15)	7/7	46	"
"	Mt	nad7	II	e/i	e	tttg-ta-tct-atccca (0)	tttgctagtcttattcca (4)	15/18	37	"
"	Mt	nad7	II	e	i	cagcc (2)	cagtc (16)	5/5	28	"
"	Mt	nad7	II	i	i/e	agccg-ct-ga (10)	acccgactaga (0)	9/11	30	"

"	Mt	nad7	II	e	i/e	accttattctgatcgttt (3)	acccta-tct-at-gttt (0)	18/15	36	"
"	Mt	nad7	II	e	i	agtggtg (15)	agtggtg (17)	7/7	46	"
"	Mt	nad7	II	e	e	ccatg--gaa (2)	ccatttcgaa (9)	8/10	29	"
"	Mt	nad4	II	e/i	e	tatgttgtgcg (0)	tatggggttcg (14)	11/11	36	"
"	Mt	nad4	II	i	i	atcttg-ct-ta (7)	atctttggctcta (0)	11/13	31	"
"	Mt	nad4	II	e/i	i/e	ttcttgtt-ggagggg (0)	tacttgttcggatggg (0)	15/16	31	"
"	Mt	ccmFc	II	i	i	tggattcggcg (4)	tgtattcggcg (10)	11/11	36	"
"	Mt	rps10	II	e/i	e	ggtttatttgcgataa (0)	ggcttttttg-aataa (10)	16/15	41	"
"	Mt	nad2	II	i	e	ttttctctatac (8)	ttttc-cttgac (1)	12/11	32	"
"	Mt	ccmFc	II	i	i	tggattcggcg (4)	tgtattcggcg (10)	11/11	36	"
"	Mt	rps3	II	e/i	i	agggcga (0)	acggcga (19)	7/7	33	"
"	Mt	trnA	I	e	e	tcgcct-gagcttggc-gact (11)	tcgcctcgaga-tggttgact (4)	19/20	54	"
"	Mt	nad7	II	i	e	gctactaggac (11)	gct-gtagcac (21)	11/10	53	"
"	Mt	nad7	II	i	e	acgagaag (21)	ac-agaag (9)	8/7	45	"
"	Mt	nad7	II	e	i	gcacaagaa (20)	gta-aagaa (28)	9/8	65	"
"	Mt	nad7	II	i	e	ggccagaaagctggt (16)	ggcgtgcaaggtggt (22)	15/15	68	"
"	Pl	trnK	I	i	e	gagta (9)	gagta (9)	5/5	28	MT120813
"	Pl	trnV	I	i	i	tgtttt (8)	tgtttt (0)	6/6	20	"
"	Pl	ndhA	II	e	i	aa-g-atc-ca (3)	aacgaatcgca (1)	8/11	23	"
"	Pl	trnA	I	i	e	ctct--caa (19)	ctctaccaa (13)	7/9	48	"
"	Pl	trnI	I	i	e	tgggccatcctgga (6)	tgggcc-tcccgga (7)	14/13	40	"
"	Pl	ycf3 (1)	II	e/i	i/e	gg-tg-cg (0)	ggatgtcg (0)	6/8	14	"
"	Pl	ycf3 (2)	II	i	i	tgcgac (3)	tccgac (0)	6/6	15	"
"	Pl	rpoC1	II	e	i	attaga (7)	attgga (12)	6/6	31	"
"	Pl	atpF	II	e	e	tgattttt (5)	tgatttatt (4)	9/9	27	"
"	Pl	trnG	I	e/i	i	taa-gggt-tatagt-cga (0)	taaggggtcgatagaacga (7)	16/19	42	"
"	Pl	clpP	II	i	e	tgagagtatacta (1)	tgagggaatgcta (4)	13/13	31	"
"	Pl	petB	II	e/i	e	tatgagtgtgt (0)	tatgattg-gt (6)	11/10	27	"
"	Pl	petD	II	i	e	tgacttgaa (5)	tgacttgaa (11)	9/9	34	"
"	Pl	rpl2	II	i	i	tagattc (8)	tag-ttc (9)	7/6	30	"
"	Pl	trnL	I	e/i	e	gact-taa (0)	gactttaa (1)	7/8	16	"
**EXCAVATA**										
uncultured trypanosome	Nu	SL-leader	Spl	e/i	i	tatattggta-tgc-gaa (0)	tattttgggagcgcggaa (8)	16/18	42	KR056281
Euglena gracilis	Pl	tufA	II	e/i	i/e	aa-gatta-aggaa (0)	aatgat-acagaaa (0)	12/13	25	Z11874
"	Pl	tufA	III	e/i	e	aaggttatacaaaataaacta (0)	aacgttacccaacataat-ta (3)	21/20	44	"
"	Pl	tufA	III	e	i	aaaaacaattacgaa (15)	aaaatcacttttaaa (17)	15/15	62	"
"	Pl	petG	II	e/i	e	ttgtgtggt-tctattt (0)	ttgtcaggtat-tattt (0)	16/16	32	"
"	Pl	psaA	II	i	i	tattttta (10)	tagtttta (1)	8/8	27	"
"	Pl	psaA	II	i	i	ttaatata (9)	ttaattttta (1)	9/9	26	"
"	Pl	psaA	II	e	e	tttacgttaatg (2)	tttat-ttagtg (1)	12/11	26	"
"	Pl	psaB	II	i	i/e	atcgtttta (5)	at-gtttta (0)	9/8	22	"
"	Pl	psaB	II	e	i/e	cttttgctca (11)	ctttaactca (0)	10/10	31	"
"	Pl	psaB	II	i	e	atttttaattt (31)	atttt-a-ttt (27)	11/9	78	"
"	Pl	psaB	II	e	i	ttacatta (4)	tta-ttta (18)	8/7	37	"
"	Pl	psaB	II	e	e	aatcctttcgg (2)	aatagttt-gg (2)	11/10	25	"
"	Pl	psbE	II	e	i/e	ttctgatatt (0)	tt-tcatatt (0)	10/9	19	"
"	Pl	psbE	II	e/i	i/e	tgt-gtgta (0)	tatagtgta (0)	8/9	17	"
"	Pl	psbF	II-t-x	e	-	5' taaa-taaaatttat (13)	-	14/-	54	"

"	Pl	psbF	II-t-n	e	i	tttatgatatgaaag (4)	tttttg-tatgaaag (21)	15/14	54	"
"	Pl	psbF	II-t-x	-	i	-	ttaattaaaatt-at 3' (13)	-/14	[54][g]	"
"	Pl	rpl23	III	i	i	cttat-attttttttttataat (7)	cttttgattttttttt-a-aat (4)	22/21	54	"
"	Pl	rpl23	III	e	i	tacttttgatgtt (12)	tactttt-atttt (22)	13/12	59	"
"	Pl	rpl23	III	i	i/e	ttatattat (6)	ttatataat (0)	9/9	24	"
"	Pl	rpl23	III	i	e	agttttact (3)	agttt-act (1)	9/8	21	"
"	Pl	rps19	III	e/i	i/e	atcgttttttga (0)	atcttttttttaa (0)	12/12	22	"
"	Pl	rps19	III	e/i	e	taggtcacttt-tg (0)	taggtgaatttgtg (0)	13/14	27	"
"	Pl	rpl22	II	e/i	i/e	tcatataactattat (0)	tcaattaaa-attat (0)	15/14	29	"
"	Pl	rps3	III-t-x	e/i	-	5' tattactattgag (0)	-	13/-	24	"
"	Pl	rps3	II-t-n	e	i	tatttata (0)	tattttta (7)	8/8	23	"
"	Pl	rps3	III-t-x	-	i/e	-	tattt-tctt-ag 3' (0)	-/12	[24][g]	"
"	Pl	rps3	III	e	i	attattttaatgatata (4)	attttattaattttata (0)	17/17	38	"
"	Pl	ORF516	II	i	i/e	tcattaattt (6)	tcattagttt (0)	10/10	26	"
"	Pl	ORF516	III	e	i	tta-aaagtttta (9)	ttatgaggtttta (15)	12/13	49	"
"	Pl	ORF516	II	e	i/e	taa-tttttaatttt (15)	tagtttttttaatttt (0)	14/15	44	"
"	Pl	ORF516	II	e	e	tta-aaagttttatt (18)	ttataag-tttatt (15)	14/14	61	"
"	Pl	rpl16	III-t-x	e/i	-	5' tctatatgagactttttttatgtaa (0)	-	25/-	52	"
"	Pl	rpl16	II-t-n	e/i	i	attatgcttgt (0)	attgtacttgt (2)	11/11	24	"
"	Pl	rpl16	III-t-x	-	i	-	tctaaaaaaaaaatttttttttaa 3' (2)	-/25	[52][g]	"
"	Pl	rpl14	III	e/i	i	ttgtaaa-att (0)	ttataaagatt (14)	10/11	35	"
"	Pl	rpl15	III	i	i	ttttattataaa (8)	ttttat-aaaaa (3)	12/11	34	"
"	Pl	rps8	III	i	i	tttttttta (6)	ttttattta (8)	9/9	32	"
"	Pl	rps8	II	e/i	i	tttaactgttg (0)	tttaa-t-tttg (7)	11/9	27	"
"	Pl	rps14	III	e	e	gaaatagtttg (12)	gaaatcgtt-g (1)	11/10	34	"
"	Pl	rps2	III	i	e	cttttt (6)	cttttt (4)	6/6	22	"
"	Pl	rps2	III	e/i	i	ttttttgataataaaaattata (0)	tttgttg-caatggggttt-ta (7)	22/20	49	"
"	Pl	rps2	III	e/i	e	aatttttat (0)	aatattttt (9)	9/9	27	"
"	Pl	rps2	II	e/i	i/e	aactata-gtgcgttattca (0)	aaat-taaatgcgt-attta (0)	19/18	37	"
"	Pl	atpI	III	e/i	i/e	aatattttttgt (0)	attttttctgt (0)	11/11	22	"
"	Pl	atpI	III	i	i/e	ttttaaaaatt (5)	ttttaaaaatt (0)	11/11	27	"
"	Pl	atpI	III	e	i/e	taagaaaattttaac-ttttaaa-aa-ttg (8)	taaaaatattttattatttttaagaagttg (0)	27/30	65	"
"	Pl	atpI	II	i	i	gttttgaatttat (4)	gtttttattat (1)	11/11	31	"
"	Pl	atpI	III	i	e	atattttattaaagaat (5)	atattta--aaa-aat (3)	17/14	39	"
"	Pl	atpI	III	e	i	aagctttaattttt (18)	aaggatt-attttt (8)	14/13	53	"
"	Pl	atpF	II	i	e	aattacttttc (22)	aat-a-tttta (13)	11/9	55	"
"	Pl	atpF	II	i	i/e	ttatttttaattttaattt (8)	ttattttta-tct-aattt (0)	19/17	44	"
"	Pl	atpF	II	e/i	e	aaca-ttttg-tgtgattcgtctatt (0)	aaaagttttgatataattcgt-tctt (5)	24/25	54	"
"	Pl	atpA	II	e	i	ttggcggaatattt (2)	tt-gagtaattatt (13)	14/13	42	"
"	Pl	atpA	II	i	e	gtaaaagtcaatattg (9)	gcacaag-cttatag (6)	16/15	46	"
"	Pl	rps18	III	i	e	ataaaaatgttaata (18)	attaaaagacaata (28)	14/14	76	"

[a] Mt = mitochondrial; Nu = nuclear; Pl = plastid

[b] Arc = archaeal; I = group I; II = group II; group III = III; twintrons (introns within introns) = II-t-x (external intron), II-t-n (internal intron) or III-t-x, III-t-n; Spl = spliceosomal; numbers in parentheses indicate intron number in a single gene. Four spliceosomal introns are given for the human dystrophin gene, which were the only readily available intron sequences for that gene in the NCBI database.

[c] e = repeat is within the exon; e/i = repeat spans the exon/intron border; i = repeat is within the intron.

[d] Red font indicates intron regions, black font indicates exon regions.

[e] Direct repeat lengths, the first number is the length of the 5' DR, the second number is the length of the 3' DR. Gaps are excluded.

[f] Search length includes the lengths of the direct repeats plus the distance to the 5' e/i and 3' i/e borders. For example, in the first row, the 5' repeat is 3 nt in length, and the 3' repeat is 4 nt in length, while both are immediately adjacent to the exon/intron orders (lengths "0" in parentheses). Therefore, the search length is 3 + 4 + 0 + 0 = 7, indicating that the direct repeats occur within a 7 nt span.

[g] Number indicates the length for the outside twintron which is joined with the 5' end of the twintron, which is already give above this number.
